# A Generic Transformation Approach for Complex Laboratory Data Using the Fast Healthcare Interoperability Resources Mapping Language: Method Development and Implementation

**DOI:** 10.2196/57569

**Published:** 2024-10-18

**Authors:** Jesse Kruse, Joshua Wiedekopf, Ann-Kristin Kock-Schoppenhauer, Andrea Essenwanger, Josef Ingenerf, Hannes Ulrich

**Affiliations:** 1 LADR Laboratory Group Dr Kramer & Colleagues, Geesthacht, Germany; 2 IT Center for Clinical Research, University of Luebeck, Luebeck, Germany; 3 Institute of Medical Informatics, University of Luebeck, Luebeck, Germany; 4 mio42 LLC, Berlin, Germany; 5 Institute for Medical Informatics and Statistics, Kiel University and University Hospital Schleswig-Holstein, Kaistraße 101, Kiel, 24114, Germany, 49 431-500-31601

**Keywords:** FHIR, StructureMaps, FHIR mapping language, laboratory data, mapping, standardization, data science, healthcare system, HIS, information system, electronic healthcare record, health care system, electronic health record, health information system

## Abstract

**Background:**

Reaching meaningful interoperability between proprietary health care systems is a ubiquitous task in medical informatics, where communication servers are traditionally used for referring and transforming data from the source to target systems. The Mirth Connect Server, an open-source communication server, offers, in addition to the exchange functionality, functions for simultaneous manipulation of data. The standard Fast Healthcare Interoperability Resources (FHIR) has recently become increasingly prevalent in national health care systems. FHIR specifies its own standardized mechanisms for transforming data structures using StructureMaps and the FHIR mapping language (FML).

**Objective:**

In this study, a generic approach is developed, which allows for the application of declarative mapping rules defined using FML in an exchangeable manner. A transformation engine is required to execute the mapping rules.

**Methods:**

FHIR natively defines resources to support the conversion of instance data, such as an FHIR StructureMap. This resource encodes all information required to transform data from a source system to a target system. In our approach, this information is defined in an implementation-independent manner using FML. Once the mapping has been defined, executable Mirth channels are automatically generated from the resources containing the mapping in JavaScript format. These channels can then be deployed to the Mirth Connect Server.

**Results:**

The resulting tool is called FML2Mirth, a Java-based transformer that derives Mirth channels from detailed declarative mapping rules based on the underlying StructureMaps. Implementation of the *translate* functionality is provided by the integration of a terminology server, and to achieve conformity with existing profiles, validation via the FHIR validator is built in. The system was evaluated for its practical use by transforming Labordatenträger version 2 (LDTv.2) laboratory results into Medical Information Object (*Medizinisches Informationsobjekt*) laboratory reports in accordance with the National Association of Statutory Health Insurance Physicians’ specifications and into the HL7 (Health Level Seven) Europe Laboratory Report. The system could generate complex structures, but LDTv.2 lacks some information to fully comply with the specification.

**Conclusions:**

The tool for the auto-generation of Mirth channels was successfully presented. Our tests reveal the feasibility of using the complex structures of the mapping language in combination with a terminology server to transform instance data. Although the Mirth Server and the FHIR are well established in medical informatics, the combination offers space for more research, especially with regard to FML. Simultaneously, it can be stated that the mapping language still has implementation-related shortcomings that can be compensated by Mirth Connect as a base technology.

## Introduction

Digitalization is progressively transforming health care systems, and its rapid pace is creating new clinical data sources that need to be integrated. This is of enormous importance for patient care, as data integration and the fusion of all sources allow a comprehensive, holistic overview and ensure the best treatment possible. However, the lack of interoperability of health care systems is a significant, long-lasting problem [1]. Nonetheless, interoperability is required to ensure seamless and effortless access to essential health care information. Existing medical data are presented in a multitude of proprietary formats and in line with different standards [2]. Therefore, it is inevitable to transform data into a harmonized structure to enable collective use. Current national and international initiatives are integrating large volumes of data in clinical environments to enable their use [3-5]. The established data integration is close to the origin of the data, which is highly desirable since late mapping harbors disadvantages and issues can be addressed accordingly [6].

Aligning the multitude of standards and thus making data usable is a major task. The mapping from source to target data structures is mainly defined by data stewards and is followed by a qualitive evaluation involving medical professionals. The involvement of these key professional groups is much easier if concise, declarative mapping rules are used instead of less comprehensible program instructions in various scripting languages. Basically, the creation of mapping rules is time-consuming and demands a significant number of resources. It occurs at every site involved, which commits a substantial additional number of stewards and medical professionals across the entire health care system [6,7]. Furthermore, sharing and reusing of declarative mapping rules in a standardized and adaptable manner is highly desirable and can release the needed resources and accelerate data enabling. Hence, this study aims to provide a software solution called “FML2Mirth,” which is based on the separation of declarative mapping rules from a transformation pipeline leveraging the functionalities of Mirth.

## Methods

The transformation of health care–related information needs two major components to enable generalization: a highly adaptable exchange format and a modular transformation engine. Our approach is based on the HL7 (Health Level Seven) Fast Healthcare Interoperability Resources (FHIR) standard and the Mirth communication server.

### Ethical Considerations

No ethics committee approval was required for this study, as the data used comprised 20 anonymized laboratory data carriers (LDT) that were provided by the laboratory company LADR Laboratory Group as part of its quality control and represent a complete blood count. According to institutional and local policies, ethics approval is not necessary for studies using anonymized laboratory testing data, and this poses no risk to individual participants.

### HL7 FHIR and the FHIR Mapping Language

HL7 FHIR is the emerging standard for health care–specific data exchange and has been broadly adapted worldwide [8]. FHIR provides state-of-the-art technologies to modernize the current health care landscape using extensible resources as harmonized and semantically annotatable information units [9]. However, FHIR also provides mechanisms to natively define transformations on its structures. The essential FHIR resource for automated conversion of instance data is the StructureMap, which defines a mapping from a source structure to a target structure [10] and provides all necessary information for an automatic transformation. To aid the definition of StructureMap resources, FHIR has specified a domain-specific, declarative language, the FHIR mapping language (FML) [11], which was introduced as part of FHIR release 3. By specifying the mappings in an implementation-independent manner, the mapping between structures can be easily shared within the medical informatics community.

One major benefit is the use of generic data structures as input formats, since FML can define mappings on non-FHIR structures. Consequently, FML can also be used to map older health care–related formats such as HL7 (version 2) or LDT to FHIR.

**Figure 1.**
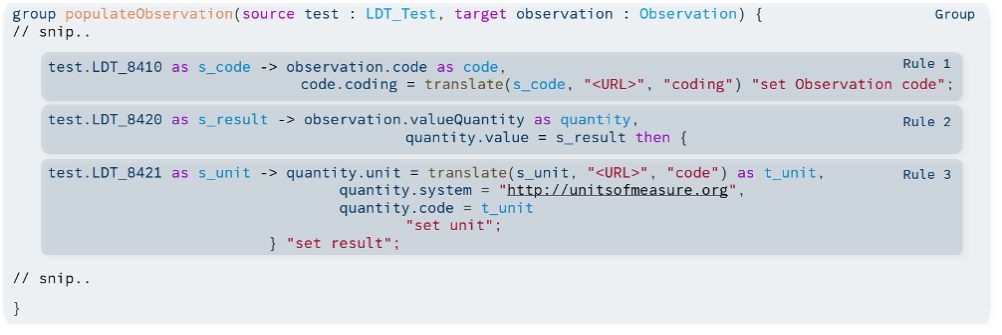
The code illustrates a snippet of the FHIR (Fast Healthcare Interoperability Resources) mapping language, showing how a conventional laboratory test in Labordatenträger format can be mapped to an FHIR observation resource. The mapping is structured in groups, containing specific rules.

The declarative syntax of FML enables users to define mappings concisely. The mapping itself is structured in so-called *groups* within the mapping, being formalized by mapping rules. These rules consist of two parts separated by an arrow. On the left side of the arrow is the source part. Here, fields of source structures can be accessed, and the values can be written in variables. On the right side, the fields of a target structure can be accessed and built-in transformation functions can be invoked (see Figure 1). Those provided functions cover a broad functionality, ranging from a simple *copy* to a *translate* function to resolve given concept codes using an external FHIR ConceptMap.

### Mirth Connect Communication Server

Mirth Connect is an open-source communication server designed specifically for the health care sector. It features support for domain-specific formats, such as HL7 (version 2), as well as more general formats such as XML and JSON. Mirth is widely used as a communication server and is applied in various areas for data integration [12,13]. As a communication server, Mirth receives messages from one system and forwards them to downstream systems. Mirth introduces the concept of *channels* to establish a connection from a source system to various target systems. Within the channels, rule-based transformers can be defined to enable message manipulation. The received message can be automatically modified in terms of content or structure. The transformer rules are implemented as JavaScript code snippets, which Mirth applies to the messages. The channels including its transformers are serialized as XML files and can easily be shared and redeployed at other sites.

### Architecture for Generic Data Transformation

**Figure 2.**
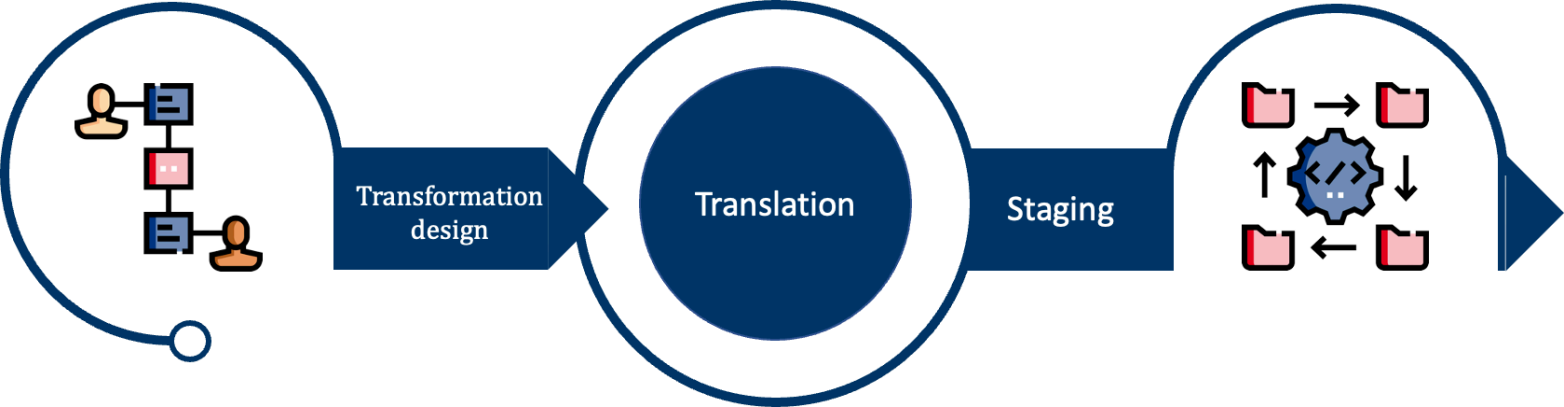
Conceptual overview of the 2-step process. In the first phase, the mapping is formalized as an FHIR (Fast Healthcare Interoperability Resources) mapping language script and corresponding semantic CodeSystems are aligned with each other in a ConceptMap. In the second phase, prior defined rules are used to deploy the mapping to Mirth. If semantic translation is needed, the ConceptMaps can be queried during run time.

The two-fold process shown in Figure 2 enables light and adaptable data transformation: the first step is transformation design and the second one is staging.

During the transformation design phase, the source and target systems are selected, and the mapping is declared in FML. The mapping needs to be created manually by a data steward. This process can be supported by previously proposed algorithms and tools [14]. In addition, semantic concepts and codes can be mapped from source to target based on prepared mapping relationships provided by the FHIR ConceptMap resource, if necessary. It is recommended that this process is carried out by a health care professional to ensure data validity and integrity.

In the staging phase, the Mirth channel, which was built on the basis of prior mapping efforts, is injected in the productive communication server at run time. Mapping within the transformer uses the rules derived from FML for format manipulation, and dynamically translates the semantic codes using the ConceptMap.

## Results

### Overview

The proposed system implements the transition between the two process phases: transformation design and staging. This transition is achieved by the in Java-implemented generator FML2Mirth [15], which derives Mirth channels from a given mapping. There are two types of files used in the process, which need to be created upfront. The first type is ConceptMaps used by a terminology server to provide a translation service for concepts that need to be translated during data transformation in the production phase. These ConceptMaps are referenced by the *translate* operations in the FML to specify how a given concept in the source structure should be mapped to a concept used in the target structure.

In the second file, the FML script, the mapping between source and target structure is defined. This script is then used as an input for FML2Mirth. As a first step, the FML script must be parsed, for which we use the official FHIR validator, since it provides functionality to generate a StructureMap from a given FML-Script. An additional advantage of this approach is, that by using a standard tool, it is guaranteed that the produced artifact is a standard-compliant StructureMap. Using these files as the input, FML2Mirth should also be compatible with every other StructureMap that adheres to the standard.

**Figure 3.**
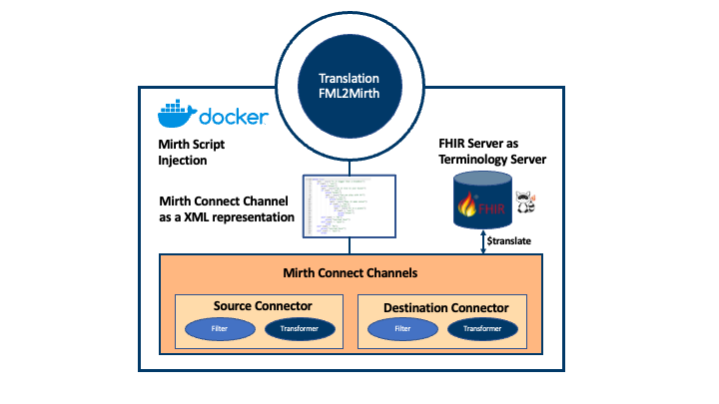
Conceptual overview of the proposed architecture for a generic data transformation process.

Besides the FML script, FHIR2Mirth needs the API (application programming interface) address of the Mirth server to which the generated channel should be deployed, as well as the address of the terminology server that is queried for the translation service during a transformation in the production phase. The tool then parses the StructureMap and generates the JavaScript code needed for every transformation specified. Once the generation is finished, the resulting code is embedded within a Mirth channel definition and deployed on the specified server via the Mirth REST-API. Subsequently, the incoming messages are automatically transformed into the desired structure. On encountering the FML *translate* function, Mirth queries the given terminology server to carry out translation between the different CodeSystems of the source and the target structure based on the previously created ConceptMaps. In our setting shown in Figure 3, an HAPI FHIR JPA server instance is used as an external terminology server, but any other FHIR terminology server implementation could be used instead. The transformed message is then forwarded to the defined target system, for example, an FHIR repository.

**Figure 4.**
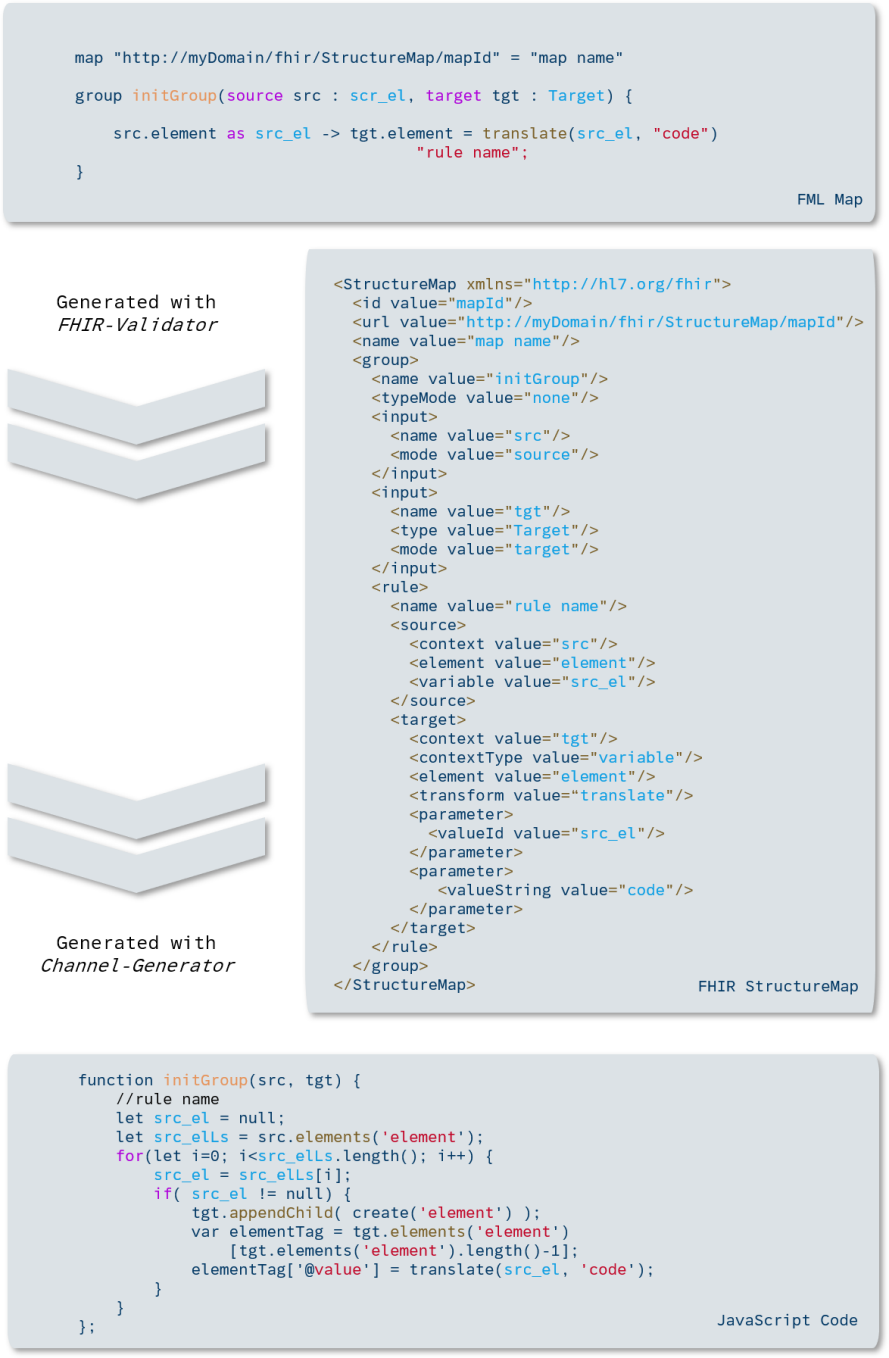
This example shows the different transformation steps, including the FHIR (Fast Healthcare Interoperability Resources) StructureMap and JavaScript result.

Evaluation of the transformation within Mirth is implemented using the JUnit testing framework. While JUnit is most commonly used for unit testing, it can also be used for end-to-end integration testing. The JUnit runner loads LDT laboratory messages from files and sends them to the Mirth server via the REST API. The destination connector of the test channel is a JavaScript connector that returns the transformed message as a response to the requesting system. The JUnit runner then receives the transformed message and uses HAPI FHIR to parse the incoming bundles as FHIR instances. Thus, the incoming resources are automatically tested for their conformity to the specification and validated against the corresponding profiles using the HAPI instance validator (Figure 4).

### Evaluation

Assessment of our proposed transformation process is carried out in cooperation with the LADR laboratory network [16], an association of 19 specialist laboratories throughout Germany serving more than 400 clinics. As an evaluation scenario, laboratory findings standardized in the German LDT [17] format are transformed into FHIR. The LDT format is a perennial standard developed and published by the National Association of Statutory Health Insurance Physicians (NASHIP; German: Kassenärztliche Bundesvereinigung [KBV]) and is used daily for communicating up to 1 million messages in Germany. In our evaluation scenario, LADR provided 20 anonymized LDT laboratory messages representing complete blood counts in LDT (version 2) according to the data format description in FHIR (version 5.12) for the evaluation. Two different FHIR implementation guides were used as target formats: the *German Laboratory finding of the NASHIP* and the *HL7 Europe Laboratory Report* [18].

The LDT format is mapped to both target formats using FML. Using the FML2Mirth generator, the FML scripts are translated into StructureMaps, and two separate channels are generated and deployed on a Mirth instance. Each format has a distinct set of semantic codes, which are permissible values for certain elements (binding). The sending system in our evaluation scenario uses proprietary codes that must be mapped to the specified CodeSystem in the target profiles. A terminology server is used to provide a translate service based on ConceptMaps, which is accessed by the Mirth channel via a REST API. The initial FML script for the transformation to the target format *MIO Laborbefund* with its 9 profiles [19] of the NASHIP consists of over 400 lines of formalized mapping rules, resulting in StructureMaps with over 3500 lines and more than 2300 elements. The 20 transformed LDT laboratory findings resulted in an average of 62 unique FHIR each. Only one report failed the closing instance validation due to a missing first name within the corresponding patient resource.

The second Mirth channel transforming LDT to the 8 profiles of the Europe Laboratory Report is based on the initial FML script. The same 20 LDT laboratory results sum up a total of over 694 FHIR resources, resulting in an average of 35 resources each. The subsequent validation process revealed for all test cases a structural issue that occurred due to a required extension of the DiagnosticReport profile. The extension should be a Composition reference to the report, but the extension itself is missing in the provided FHIR package by EU (European Union) laboratory report, and the referral link is nonfunctional. Yet, a manual check and a comparison with the NASHIP MIO resources proved their validity.

## Discussion

This study presents a concept and the implementation of an adaptable transformation process for various data structures into a standardized format. Our approach is based on FHIR as a standardized data exchange format, FML as declarative transformation rules, and Mirth Connect as the transformation executor.

### Principal Results

The approach focusses on separating the transformation rules from the actual transformation to extract the rules out of the process. Due to the detachment, the rules, which are created in a labor-intensive process, can be shared in a declarative and standardized manner. The use of FML for defining the mappings can cover technical operations such as network calls to the terminology server and are triggered by FML2Mirth dynamically during JavaScript generation. In addition, the separation allows a degree of modularization and, thus, the reuse of the created rules. For example, it was possible to modify the mapping for the MIO reports to generate laboratory reports, which conform to the profiles of the EU laboratory report within a few hours. This emphasizes the flexibility of the proposed method. A significant advantage of this approach is that it allows mappings of concepts to be exchanged at run time of the Mirth channel and be developed and updated independently of the structural mapping. Compared to common ETL jobs, this approach needs much less manual intervention.

The mapping is defined using FML to render the StructureMap using the official FHIR Validator. The declarative mapping is enclosed in the generated StructureMap instances as a parse tree. Referring and combining various FML scripts is possible per the specification but has not been implemented yet. Therefore, the mapping shall be edited incrementally during the transformation design phase in the FML scripts rather than in the StructureMaps directly. Nevertheless, initial creation of the FML scripts is less time-consuming but still a labor-intensive task, and currently there is a lack of suitable tool support. An FML script is always a 1-way mapping, and reverse transformation is not automatically assumed.

The functionality provided by Mirth highly constrains the design of our approach, as external dependencies should be minimized to ensure ease of use. With this in mind, the implemented single command line tool in Java injects the transformations into a specified Mirth channel. In addition, only the Mirth server and an FHIR server, which provides terminology services, are required. Simultaneously, the system should be as flexible as possible and support arbitrary StructureMaps. To fulfill these requirements, it was necessary to work with the circumstances provided by Mirth. Since Mirth itself works on XML objects, it is prudent to use this approach as well. Hence, the transformations defined in the StructureMap are translated into transformations on XML objects. Mirth itself first translates incoming data such as HL7v2 to XML as a first processing step, so all formats supported by Mirth can automatically also be transformed using the generated transformer from FML2Mirth. If a format is not supported by Mirth, such as LDT, another transformation step can simply be added to transform the incoming data into an XML structure.

### Limitations

During the evaluation, an issue was discovered with the FHIR implementation within the different FHIR servers. In particular, the issue concerned FHIRPath implementation, which is used by the FHIR Validator to evaluate the validation rules. In our test setting, one rule is interpreted differently depending on the implementation used. On the tested laboratory reports, the .NET and JavaScript implementations returned true, which is correct per our understanding. In contrast, both Java implementations tested (HAPI and IBM) returned false.

Alongside the complex structural mapping, semantic integrity must also be ensured. In our approach, the semantic mappings are created and validated by health care professionals and stored in an FHIR server as an external service, which is standard procedure. While existing terminology systems generally provide transition rules to newer versions, the maintenance of FHIR resources is not standardized and is an ongoing topic of investigation in medical informatics [20].

Furthermore, a mechanism was missing to process further StructureMaps, which are referenced from a given StructureMap. FML uses FHIRPath as an embedded language in several instances. The associated specification is extensive and was implemented in this study only in parts.

### Comparison With Prior Work

The use of formalized mapping rules in data integration is a well-studied topic, and Mirth as a transformation engine for clinical data integration is also a renowned tool. However, the combination of both topics with the goal of a generic and easily adaptive mapper is missing in the literature, to our knowledge. Alongside declarative rules, the work of Ong et al [21] must be mentioned; they present a dynamic ETL approach that uses a custom mapping language to transform health care–related data into the OMOP (Observational Medical Outcomes Partnership) common data model. The formalized rules are rich in details but are proprietary and rather database-oriented due to their use case. An innovative feasibility study for FML has been presented by Dimitrov et al [22]; they used FML for transforming the HL7 CDA (Clinical Document Architecture)–based national Austrian electronic patient record. Using FML, a transformation from CDA documents to the International Patient Summary based on FHIR could be accomplished. The usage of Mirth as a transformation engine is an intuitive choice due to its functionality and product scope. Hence, various studies are focusing on the transformation from HL7 (version 2 ) messages into further target formats, including structural reformation into JSON [23] or standardization into HL7 FHIR [24].

Our approach is based on that of a prior study that evaluated the transformation of StructureMaps into Mirth channels [25]. The results were promising, but it also emerged that the manual definition of StructureMaps became cumbersome and rapidly very complex. The presented approach overcomes this shortage using FML and its intuitive nature.

### Conclusions

We successfully implemented a functional system based on Mirth, which automatically generates transformations from StructureMaps and integrates them into a Mirth channel. This system supports large parts of the FML specification. Initial tests revealed that complex structures based on FML maps, generated in a channel in conjunction with a terminology server and ConceptMaps are feasible. If a new format is to be supported, it may only be necessary to insert another Mirth transformation step before the transformation from the StructureMap, to transform the input structure into XML. One advantage of the current solution is its flexibility and robustness in that only one resource is used for translation. The topic of FML is still an ongoing discussion and has not been extensively investigated in the FHIR community so far. Accordingly, the tooling in this area is currently rather rudimentary.

## Abbreviations

CDA: Clinical Document Architecture
ETL: Extract, Transform, Load Process
EU: European Union
FHIR: Fast Healthcare Interoperability Resources
FML: FHIR Mapping Language
IPS: International Patient Summaries
LDT: Labordatenträger
MIO: Medical Information Object (*Medizinisches Informationsobjekt*)
NASHIP: National Association of Statutory Health Insurance Physicians
OMOP: Observational Medical Outcomes Partnership
